# Biofunctional Attributes and Storage Study of Soy Milk Fermented by *Lactobacillus rhamnosus* and *Lactobacillus helveticus*

**DOI:** 10.17113/ftb.57.03.19.6103

**Published:** 2019-09

**Authors:** Birendra Kumar Mishra, Subrota Hati, Sujit Das, Jashbhai B. Prajapati

**Affiliations:** 1 Department of Rural Development and Agricultural Production, North-Eastern Hill University, 794001 Tura Campus, Meghalaya, India; 2 Department of Dairy Microbiology, SMC College of Dairy Science, Anand Agricultural University, 388110 Anand, Gujarat, India

**Keywords:** soy yoghurt, ACE inhibitory activity, antioxidant activity, antimicrobial activity, isoflavones, *Lactobacillus* sp

## Abstract

Different soybean products are considered as traditional functional food among the Mongolian population in Northeast India. In the present study, the effect of different flavours (mango, orange, vanilla and white rose), inoculation rates of *Lactobacillus rhamnosus* K4E (KX950834) and *Lactobacillus helveticus* K14 (KU644578), and mass fractions of skimmed milk and sugar on the acceptability of soy yoghurts was studied. Physicochemical (pH, titratable acidity) and microbial analyses (total bacterial, total coliform, yeast and mould count) were conducted, and organoleptic (aroma, taste, colour, mouthfeel, texture and overall acceptability) and biofunctional properties (angiotensin-converting enzyme (ACE) inhibitory activity, antioxidant and antimicrobial activities and biotransformation of isoflavones) were evaluated during storage up to 10 days under refrigeration conditions (6–8 °C). Panellists preferred white rose soy yoghurt more than other flavours. The pH was from 5.65 to 4.20, the titratable acidity (expressed as mass fraction of lactic acid) was from 0.33 to 0.51% and total *Lactobacillus* count ranged from 6.81 to 8.69 log CFU/mL during storage. The ACE inhibitory activity increased from 21.17% on day 0 to 81.03% on day 5, followed by a decrease of the activity after 10 days (38.85%). The antioxidant activity was the highest on day 5 (87%). White rose soy yoghurt had the highest antimicrobial activity against *Listeria monocytogenes,* followed by *Bacillus subtilis, Staphylococcus aureus, Salmonella typhi* and *Escherichia coli*. RP-HPLC analysis showed that after 18 h, the production of soy isoflavone aglycones genistein and daidzein in yoghurt was 87.3 and 58.4%, respectively.

## INTRODUCTION

Through the variety of traditional indigenous foods of different ethnic communities like Khasi, Jaintia, and Garos, it can be stated that Meghalaya is indeed a biodiversity-rich area of the northeastern region of India. As an economical source of plant protein, various fermented soybean foods can be considered on a par with animal and milk products based on protein cost per kg, which can be easily accessed by the rural population residing in that region (*1*). There has always been an increasing interest for the utilization of *Lactobacillus* strains obtained from traditional/natural fermented dairy products on a commercial scale, but limited knowledge has been obtained related to the microbiota of these products (*2*, *3*). The people of Meghalaya have their very own specific dietary/food consumption pattern when it comes to traditional fermented soy foods, namely ’tungrymbai’ of Khasi tribe, ’hawaijar’ of Meitei tribe, ’bekang’ of Mizo tribe, ’aakhone’ of Naga tribe and ’peruyaan’ of Apatani tribe are a few of the traditional fermented soy foods belonging to various states in the north-eastern region of India (*4*).

Lactobacilli require the free amino acids during their growth and they release them during fermentation of milk or other proteins and produce smaller peptides, which help them to grow. Typical beany flavour of soy milk can get transformed into aromatic compounds that could improve the flavour of yoghurt during the fermentation of soy milk by the lactic acid bacteria (LAB) (*5*). Soy milk yoghurts have emerged as a popular alternative to traditional dairy-based yoghurts because they reduce the level of cholesterol (0 mg/L), saturated fat (0.2 g/L), and lactose (0 g/L) (*6*). Fermented soybean is a precious source of angiotensin-converting enzyme (ACE) inhibitor (*7*). Bioactive peptides with high oxidant potential are formed during the enzymatic hydrolysis or microbial proteolytic activity in fermented soy milk (*8*). Several LAB species (*L. rhamnosus*, *L. helveticus*, *L. fermentum*, *L. plantarum, etc*.) in fermented food and yoghurt starter cultures possess antioxidant activity *in vitro* and *in vivo,* inducing the antioxidant defence mechanism in the host (*9*).

There is a problem with malnutrition, and protein and calorie deficiency in Meghalaya region, so fermented soy foods can act as promising supplements since the fermentation with LAB improves the bioavailability of isoflavones. Generally, isoflavone glycosides are not absorbed intact across the enterocyte but with the help of the LAB they are hydrolyzed by intestinal β-glycosidase to release the aglyconic forms: genistein, daidzein and glycitein for uptake to the peripheral circulation (*10*). Soy isoflavones have also been reported to relieve menopause symptoms and have therapeutic effects on osteoporosis (*11*, *12*). Probiotics limit the growth of the intestinal pathogens in the gastrointestinal tract by releasing various antibacterial elements like H_2_O_2_, CO_2_, diacetyl, bacteriocins, and lactic and acetic acids. They compete for nutrients and adhesion sites, increase or decrease enzyme activity, and simultaneously increase the level of antibodies and reduce macrophage activity (*13*), thus further improving microbiological quality and shelf life of various fermented food products.

The tribal population of Meghalaya suffers from lactose intolerance, so we have studied indigenous *Lactobacillus* strains with rich probiotic potential (*3*) isolated from the ethnic fermented foods of Garo Hills, Meghalaya to develop soy yoghurt. Hence, the population of this region can benefit from consuming such typical traditional soybean flavoured products with enriched biofunctional properties.

## MATERIALS AND METHODS

### Bacterial strains, growth conditions and sample preparation

The cultures used in the study, namely *Lactobacillus rhamnosus* K4E and *Lactobacillus helveticus* K14 (NCBI GenBank Accession No. KX950834 and KU644578), were isolated from the various ethnic fermented foods of Meghalaya, India, and deposited in Animal Science Laboratory, Department of Rural Development and Agricultural Production, North-Eastern Hill University, Tura Campus, Meghalaya, India (*14*). These isolates were propagated in De Man, Rogosa and Sharpe (MRS) agar; HiMedia, Mumbai, India) for 24 h at 37 °C. Their probiotic potential was studied earlier and they were selected for this study due to their maximum probiotic characteristics (*3*). Small, smooth yellow seeds of a local variety of soybeans were obtained from a local market in Tura town (West Garo Hills, Meghalaya). The pure lactic cultures (*L. rhamnosus* K4 and *L. helveticus* K14) grown in MRS were transferred into sterile rehydrated skimmed milk and glycerol (both HiMedia), and 1-mL aliquots were placed in cryovials and stored at −20 °C. After two successive transfers of the test organisms in MRS broth (HiMedia) and incubation at 37 °C for 24 h, each activated culture was inoculated into skimmed milk and incubated at 37 °C for another 16 h. These cultures were then transferred into soy milk to check their activity (*8*).

### Preparation of functional soy yoghurt with optimal composition

Soy milk was prepared according to the procedure of Hati *et al.* (*8*), with some modifications. A mass of 500 g of soybeans was soaked for 18 h in 1 L of distilled water at room temperature (28 °C). The soaking water was drained, and soybeans were blanched in boiling distilled water at 98 °C for 20 min. The beans were dehulled thoroughly by rubbing between the palms of the hand for three times to remove the testa (seed coat). The dehulled soybeans were blended (Classic WL1606 blender; Philips, Vadodara, India) with boiled distilled water (2.5 L) in a ratio of 1:5. The resulting slurry was further filtered through four layers of muslin cloth to discard the okara (filtrate). Around 2.5 L of soy milk were obtained per 500 g of soybeans and autoclaved at 121 °C for 20 min, followed by cooling to 42 °C. The soy milk was divided into eight equal parts (300 mL each) for different sample batches (Table 1).

**Table 1:** Formulation of different batches of soy yoghurt

Batch	*w*(sugar)/ %	*w*(skimmed milk powder)/ %	Food-grade flavour type	*w*(food-grade flavour)/ %	*φ*(*Lactobacillus* culture)/ %	*V*(soy milk)/ L
1	7	2	mango	0.1	2	300
2	8	3	mango	0.2	2	300
3	9	4	orange	0.1	3	300
4	10	4	orange	0.2	3	300
5	10	5	vanilla	0.2	4	300
6	10	5	white rose	0.2	4	300
7	11	6	vanilla	0.3	5	300
8	11	6	white rose	0.3	5	300

The *Lactobacillus* culture consists of *L. rhamnosus* and *L. helveticus* in 1:1 ratio

To determine the optimal soy yoghurt composition, eight batches were inoculated with *Lactobacillus* culture (*L. rhamnosus* K4E and *L. helveticus* K14) and different mass fractions of skimmed milk powder, sugar and food-grade flavours (Marson, Mumbai, India) were added (Table 1).

The eight different samples of functional soy yoghurt were used in sensory evaluation (Table 2) by a trained panel of 10 panellists (6 male and 4 female, aged 22 to 45) for selecting the specific yoghurt sample for further shelf life and biofunctional studies. The panellists had previous experience in dairy product evaluation. The panel comprised postgraduate students and faculty members of the Department of Rural Development and Agricultural production of North-Eastern Hill University, Tura Campus, Meghalaya, India. Parameters such as aroma, taste, colour, mouthfeel, texture and overall acceptability of the product were evaluated using a standard 9-point hedonic scale (9=like very much to 1=dislike very much) (*15*). Out of these eight samples, only one batch was selected by the panellists for further study of shelf life for 1, 3, 7 and 10 days under refrigeration conditions (6–8 °C) and biofunctional properties.

**Table 2:** Sensory scores of different white rose flavoured soy yoghurt batches used for the selection of optimal yoghurt formulation

Parameter	Batch							
1	2	3	4	5	6	7	8
Aroma	(3.64±0.101)^a^	(3.49±0.106)^a^	(5.00±0.085)^c^	(5.06±1.210)^c^	(4.21±0.262)^b^	(5.44±0.501)^c^	(6.18±0.552)^d^	(7.12±0.680)^e^
Taste	(4.06±0.150)^b^	(4.12±0.151)^b^	(4.83±0.125)^b^	(4.50±0.661)^b^	(4.13±0.520)^b^	(5.11±0.740)^c^	(6.11±0.056)^d^	(7.00±0.811)^e^
Colour	(4.11±0.172)^b^	(5.16±0.174)^c^	(4.44±0.874)^b^	(4.00±0.440)^b^	(4.06±0.403)^b^	(5.66±0.472)^c^	(6.05±0.224)^d^	(6.26±0.252)^d^
Mouthfeel	(3.32±0.091)^a^	(5.25± 0.092)^c^	(4.20±0.220)^b^	(5.66±0.370)^c^	(5.88±0.551)^c^	(5.70±0.055)^c^	(5.90±0.470)^c^	(7.06±0.334)^e^
Texture	(4.47±0.144)^b^	(5.32±0.147)^c^	(4.11±0.335)^b^	(5.16±0.348)^c^	(5.44±0.050)^c^	(5.35±0.420)^c^	(6.20±0.115)^d^	(6.50±0.440)^d^
Overall acceptability	(4.01±0.221)^b^	(5.11±0.224)^c^	(4.88±0.116)^b^	(5.05±0.217)^c^	(5.16±0.228)^c^	(5.88±0.505)^c^	(5.92±0.374)^c^	(7.33±0.177)^e^

Description of batches is given in Table 1. Values are mean±S.D. of eight independent determinations of each sample. Values with different letters in superscript in each column differ significantly (p<0.05)

### Assessment of acidity of white rose soy yoghurt

A digital pH meter (model no. 112; Electronics India, Parwanoo, India) was used to measure the pH of the white rose soy yoghurt samples at 25 °C after being calibrated with freshly prepared pH=7.0 and 4.0 standard buffers (Sisco Research Laboratories, Mumbai, India). The titratable acidity was evaluated as per Abraham *et al*. (*16*). A volume of 10 mL of the sample and 10 mL of distilled water were titrated with 0.1 M NaOH (Nice Chemicals, Cochin, India) using 0.5 mL phenolphthalein (Nice Chemicals) as an indicator. Titration continued and it reached endpoint when the colour changed to pink. The test was repeated thrice to get an average result (*16*) using the following equation:

Titratable acidity=(*V*_NaOH_·*c*_NaOH_·*M*_lactic acid_·100)/(*V*_sample_·1000) /1/

where *c*_NaOH_=0.1 mol/L and *M*_lactic acid_=90 g/mol.

### Microbial analysis of white rose soy yoghurt

The viability of bacteria is of major importance for providing health benefits. To determine viable cell counts, 1 mL of the yoghurt sample was diluted with 9 mL of physiological saline solution (*V*/*V*) and then serial dilutions were prepared in a ratio 1:10. An aliquot of 1 mL of different dilutions (10^–5^ and 10^–6^) of samples stored up to 10 days under refrigeration conditions (6–8 °C) was used to determine the total viable count per mL of specific growth media. The growth rate of *Lactobacilli* was studied on MRS agar. To test the presence of contaminants in the samples, coliforms were counted on eosin methylene blue agar (EMB; HiMedia) and yeasts and moulds on potato dextrose agar (PDA; HiMedia), and the viable cell count was expressed in log CFU/mL (*16*).

### Determination of ACE inhibitory activity (in vitro study)

The ACE inhibitory activity of the white rose soy yoghurt was estimated spectrophotometrically as per the method of Donkor *et al*. (*17*) with a few modifications. Inhibition was measured spectrophotometrically using UV-Vis double beam spectrophotometer (model no. 220; Systronics, Ahmedabad, India) at 228 nm and calculated using the following equation:

ACE inhibitory activity= ((*A*_control_– *A*_sample_)/*A*_control_)·100 /2/

where *A*_control_ is the absorbance of the solution without ACE inhibitory component (control) and *A*_sample_ is the absorbance of the solution with ACE and ACE inhibitory component (yoghurt sample).

### Antimicrobial activity of white rose soy yoghurt

The antimicrobial activity of the filtrate of the white rose soy yoghurt was tested by the agar well diffusion method against *Listeria*, *Salmonella typhi*, *Bacillus subtilis, Escherichia coli* and *Staphylococcus aureus.* The test microorganisms were obtained from the Dairy Microbiology Department, Anand Agricultural University, Anand, Gujarat, India. The presence of a clear zone surrounding the agar wells indicated the inhibition activity of the culture filtrates of the *Lactobacillus* isolates against the indicator bacteria (*19*).

### Determination of total antioxidant activity of white rose soy yoghurt

The antioxidant activity was measured as total radical scavenging capacity based on the ability of the white rose soy yoghurt to scavenge the stable 2,2'-azino-bis(3-ethylbenzothiazoline-6-sulphonic acid (ABTS) radical (Sigma-Aldrich, Merck, Mumbai, India) in 5 min, as per the method of Subrota *et al*. (*18*). By analyzing the reduction in the absorption at various time intervals, the antioxidant capacity of the fermented white rose soy yoghurt was determined using the following equation:

Antioxidant activity= ((*A*_C_–*A*_T_)/*A*_C_)·100 /3/

where *A*_C_ and *A*_T_ are the absorbance of the ABTS^+^ and of the tested samples, respectively.

### Estimation of bioconversion of isoflavones during fermentation

The isoflavones were extracted from white rose soy yoghurt according to the method of Otieno *et al*. (*21*) with a few alterations. For extraction of isoflavones, 10 g of frozen unfermented soy milk (control) and fermented white rose soy yoghurt prepared as described above were freeze-dried. The freeze-dried sample (0.5 g) was dissolved in 25 mL of methanol (HPLC grade) and vortexed vigorously for 10 min, then transferred into a 150-mL flask and placed in a water bath for 20 min. The mixture was poured in a 20-mL centrifuge tube (Tarsons, Kolkata, India) and centrifuged (model no. CM-12PLUS; Remi Elektrotechnik, Vasai, India) at 8000× *g* for 30 min at 4 °C. A volume of 50 µL of internal standard flavone solution (0.2 mg/mL) was added to 2-mL aliquot of the supernatant that was obtained after centrifugation and evaporated to dryness using nitrogen gas connected with refrigerated trap of the vacuum pump. Dried sample was dissolved in 1 mL of the mobile phase, containing 0.05% trifluoroacetic acid (TFA) in 50% methanol and 50% ammonium acetate (100 mM), vortexed vigorously for 10 min, then centrifuged at 15 000×*g* for 15 min using a cooling centrifuge system (Eppendorf AG 22331; Hamburg, Germany). The resulting supernatant was passed through a 0.22-µm syringe filter and 20 µL of it were further injected into the HPLC system (*20*).

The isoflavones were separated and quantified in a HPLC system (model no. RID-10A; Shimadzu, Tokyo, Japan) equipped with wavelength detector (SPD-20A), ultraviolet (UV-260 nm) detector, Shimadzu LC-20 manual injector with 20 mL loop and SeQuant® ZIC®-cHILIC PEEK coated HPLC column (250 mm×4.6 mm, 3 µm with 100 Å pore size; Merck, Darmstadt, Germany), as per the method of Hati *et al*. (*20*). The isoflavones were eluted by an isocratic flow of 30 min with mobile phase consisting of 0.05% TFA in 50% 100 mM ammonium acetate and 50% methanol at a flow rate of 1 mL/min and column temperature of 25 °C. Before connecting the mobile phase to the HPLC system, it was filtered through a 0.45-µm membrane filter (Millipore, Bengaluru, India) followed by degassing in an ultrasonic water bath (model no. LMUC-9; Labman, Chennai, India) at 37 °C for 35 min. The volume of 20 µL of supernatant of white rose flavoured soy yoghurt was then injected into the HPLC for further analysis.

### Statistical analysis

All the data presented here are the average values of three independent assays and the results obtained were expressed as mean value±standard deviation. One-way analysis of variance (ANOVA) was applied and comparison was made through Bonferroni’s test with a least significant difference at p≤0.05 using the IBM SPSS Statistical program v. 20 (*22*).

## RESULTS AND DISCUSSION

### Organoleptic properties of selected soy yoghurt during storage

The popularity of soy yoghurt as a food basically depends on its sensory characteristics. The addition of various flavours to yoghurt increases the options for consumers, and thereby helps in marketing promotion of yoghurt and retaining consumer interests as well (*23*). Based on organoleptic evaluation (Table 2) by the panellists, out of the eight batches, batch 8 had all sensory scores >6 on the hedonic scale, therefore, it was selected for further shelf life study. Batches 1 to 7 had loose body and sensory scores of texture ≤6, thus were not acceptable for further evaluation. Also, the decrease in pH and increase in titratable acidity are desired yoghurt characteristics, which those batches lacked.

To obtain desired quality of soy yoghurt, moisture and total solid content were adjusted with the addition of different mass fractions of skimmed milk powder. The percentage of skimmed milk was increased from 2 to up to 6%, as per the suggestions by the panellists, to obtain proper body, texture and acidity of yoghurt, as soy milk does not contain lactose like bovine milk. Raw soy milk as such is not preferred due to the presence of unpleasant off-flavours (beany) from soybeans and different antinutritional factors, like phytic acid, oligosaccharides, trypsin inhibitor, so to mask up the beany flavour, vanilla, white rose, mango and orange flavours were added. White rose flavour was selected as the best by the panellists during organoleptic evaluation. According to them, 5% inoculum of *Lactobacillus* culture, 6% skimmed milk powder, 11% sugar and 0.3% white rose flavour were optimal for the preparation of soy yoghurt with enhanced flavour. The optimized product was accepted by eight panel members and was used for further studies of shelf life up to 10 days. The sensory scores were dropping after day 7, followed by the growth of yeasts on the last day of storage.

White rose flavoured soy yoghurt had all sensory scores >6 after 10 days of storage at refrigeration temperature. The scores of aroma, taste, colour, mouthfeel, texture and overall acceptability on days 1, 4 and 7 significantly differed from that on day 10 (Table 3). The highest score for aroma was 7.8±0.9 on day 4, which decreased to 6.4±0.7 on day 10. The highest taste score was 7.4±0.2 on day 4, which decreased to 6.2±0.4 on the 10th day of storage. Colour, as the main parameter for the acceptance of food, scored 7.4±0.7 on day 4 and 6.7±0.3 on the final day of storage. The mouthfeel property was 7.4±0.9 on day 4 and 6.9±0.6 on day 10 of storage. The score for texture was 7.6±0.9 on day 4, which decreased to 6.5±0.5 on day 10. Overall acceptability had scores of 7.4±0.6 and 6.3±0.5 on days 4 and 10 of storage, respectively. Hence, it can be concluded that the sensory scores for all the sensory parameters were the highest on day 4 and started decreasing slightly from day 7, but still had considerable acceptability (>6). Our study is in accordance with the results of Junaid *et al*. (*24*), who reported the moderate decrease of sensory parameters of flavoured probiotic acidophilus milk after 6 days of storage, although it was still considered acceptable. The use of flavours in soy yoghurt improved the acceptability and sensory attributes and reduced the intensity of off-flavours under refrigerated conditions (*25*). Similarly, Park *et al*. (*26*) reported that the soy yoghurt produced from a mixed culture of *Lactobacillus* sp. and *Streptococcus* sp. had a higher organoleptic quality than the soy yoghurt made from a single *Streptococcus thermophilus* culture.

**Table 3:** Sensory scores of optimized white rose flavoured soy yoghurt during storage for 10 days

Parameter	*t*(storage)/day	Sensory score
Aroma	1	(7.3±0.7)^a^
	4	(7.8±0.9)^d^
	7	(7.4±0.2)^c^
	10	(6.4±0.7)^b^
Taste	1	(7.3±0.5)^a^
	4	(7.4±0.2)^c^
	7	(7.2±0.6)^a^
	10	(6.2±0.4)^b^
Colour	1	(7.0±0.7)^b^
	4	(7.4±0.7)^c^
	7	(7.2±0.8)^a^
	10	(6.7±0.3)^b^
Mouthfeel	1	(7.3±0.1)^a^
	4	(7.4±0.9)^c^
	7	(7.2±0.7)^a^
	10	(6.9±0.6)^b^
Texture	1	(7.3±0.1)^c^
	4	(7.6±0.9)^d^
	7	(7.2±0.7)^a^
	10	(6.5±0.5)^b^
Overall acceptability	1	(7.1±0.9)^a^
	4	(7.4±0.6)^c^
	7	(7.2±0.7)^a^
	10	(6.3±0.5)^b^

Values are mean±S.D. of triplicate determinations. Values with different letters in superscript in each column differ significantly (p<0.05)

### Acidity of white rose flavoured soy yoghurt

The pH of white rose soy yoghurt gradually decreased while the titratable acidity increased during storage under refrigeration conditions (6–8 °C) (Table 4). These changes in the pH and titratable acidity of the soy yoghurt occur because of the activity of *Lactobacillus* culture (*L. rhamnosus* and *L. helveticus*). The pH on day 1 differed significantly from the rest of the storage days (4, 7 and 10). The mean pH values (5.65±0.01 on day 1 to 4.20±0.02 in day 10) of all the samples decreased considerably during storage. Furthermore, the pH of the yoghurt is directly proportional to the release of nutritional metabolites, particularly lactic acid or other organic acids produced by the lactic strains (*27*). The pH values of the white rose flavoured soy yoghurt in our study were comparable to those reported by Lee *et al*. (*28*) and Osundahunsi *et al*. (*29*). The lactic acid production in the soy yoghurt was considered significant on days 4 and 7, but differed significantly from days 1 and 10. The titratable acidity ranged from (0.33±0.04) to (0.51±0.06) % from day 1 to day 10. The increase in the lactic acid content throughout the ten days of storage further confirmed the role of the *Lactobacillus* culture in the lactic acid fermentation under refrigerated conditions. In agreement with the findings of Salwa and Diekmann (*30*), and Rashid and Thakur (*31*), increased production of lactic acid decreases the pH of the product.

**Table 4:** Physicochemical analysis of optimized white rose flavoured soy yoghurt during storage

*t*(storage)/day	pH	*w*(lactic acid)/%
1	(5.65±0.01)^a^	(0.33±0.04)^a^
4	(4.54±0.05)^b^	(0.46±0.03)^b^
7	(4.37±0.02)^b^	(0.48±0.02)^b^
10	(4.20±0.02)^b^	(0.51±0.06)^c^

Values are mean±S.D. of three independent determinations of each sample. Values with different letters in superscript in each column differ significantly (p<0.05)

### Viable cell counts in white rose soy yoghurt

**Fig. 1:**
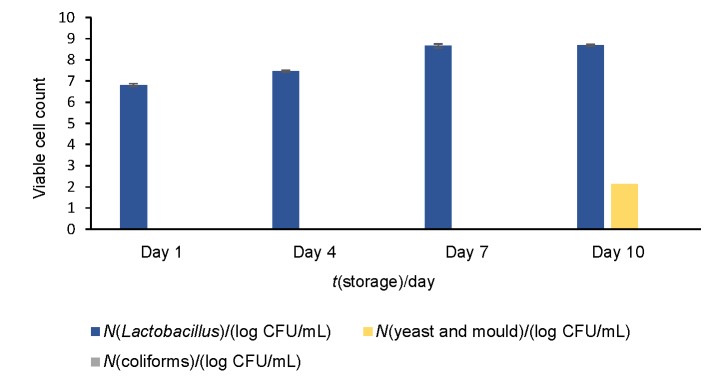
Microbial analysis of white rose flavoured soy yoghurt during storage for 10 days. Error bars show standard deviation (*N*=3); p<0.05

The microbiological evaluation was done to examine the survival of starter organisms and the presence of undesirable spoilage and pathogenic organisms in the finished product. The *Lactobacillus* counts increased during the storage from (6.81±0.07) on day 1 to (8.66±0.09) log CFU/mL on day 7. On day 10 viable cell count was almost the same ((8.69±0.03) log CFU/mL) as on day 7, which may be due to the exhaustion of nutrients for *Lactobacillus* culture during storage (Fig. 1). On days 1 and 4 the cell count of (6.81±0.07) and (7.47±0.05) log CFU/mL, respectively, was significantly different from days 7 and 10, with values (8.66±0.09) and (8.69±0.03) log CFU/mL, respectively. No coliform counts were observed during storage (Fig. 1), which signifies that the products were free from faecal contamination. Yeast and mould growth was observed after day 7, with (2.1±0.1) log CFU/mL on the last day (10) of storage, which may be a result of contamination or inefficient pasteurization of soy milk. Our study is in agreement with that of Osundahunsi *et al.* (*29*), where the microbial count in soy yoghurt of various formulations was within the acceptable range. Adams and Moss (*32*) stated that a typical yoghurt must contain more than CFU∙10^8^/g of the starter culture with <1 coliform per g, <1 mould per g and 0 yeast per g. According to Ishibashi and Shimamura (*33*), the beneficial effects of *Lactobacillus acidophilus* can be expected only after ingesting significant count of viable cells that possess the quality to colonize the human gut. Hence, white rose flavoured soy yoghurt fermented with *Lactobacillus* culture is accepted based on both physicochemical and microbial quality.

### ACE inhibitory activity of white rose flavoured soy yoghurt

**Fig. 2:**
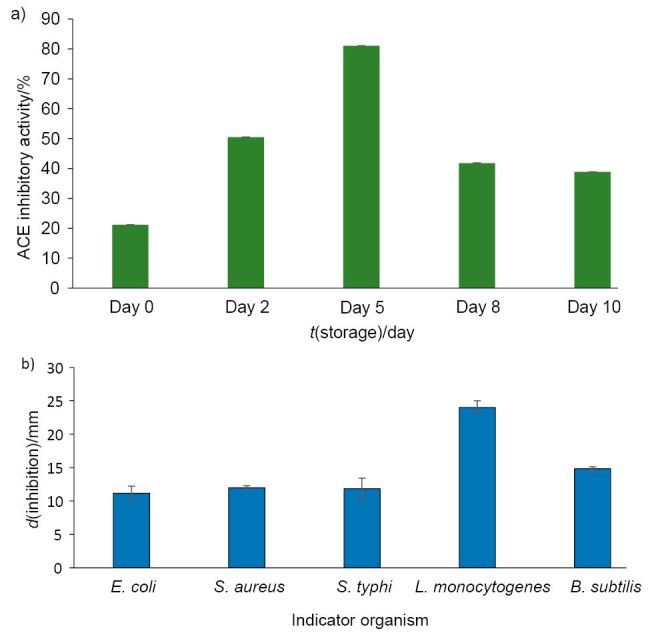
Activities of white rose flavoured soy yoghurt fermented by *Lactobacillus cultures* during 10 days of storage: a) ACE inhibitory activity, with the highest ACE inhibition clearly visible on day 5, and b) antimicrobial activity against different test organisms. Error bars show standard deviation (*N*=3); p<0.05

The analysis of ACE inhibitory activity of antihypertensive peptides derived from soy milk proteins is one of the most common methods to assess the biofunctionality of a fermented dairy product. The ACE inhibitory activity of the white rose flavoured functional soy yoghurt increased from 21.17% on day 0 to 81.03% on day 5, followed by a decrease from day 8 (41.8%) to day 10 (38.85%) as shown in Fig. 2a. Significant variation (p<0.05) in ACE inhibitory activity was observed during different storage periods. Hence, this study suggests that extremely potent ACE inhibitory peptides were produced on day 5 during storage at 6 °C. However, the values decreased sharply between days 8 and 10, indicating further proteolytic degradation of inactive peptides. It has been reported previously that fermented soy milk produced by a mixed culture of lactic acid bacteria might contain a wider variety of functional ACE inhibitory peptides than milk cultured with a single strain (*32*, *33*). Fuglsang *et al*. (*7*) reported that probiotic yoghurt contained potent ACE inhibitory peptides on day 1. During the first week of storage, ACE inhibitors seemed to be more hydrolyzed (IC_50_ values increased) on day 7, but the IC_50_ values decreased (p<0.05) sharply between days 7 and 21, indicating further proteolytic modification of inactive peptides or the release of new peptides from caseins. Hence, it can be interpreted that the LAB are capable of producing ACE inhibitors in different amounts during milk fermentation, which is in agreement with our study. Montville and Kaiser (*34*) also reported that ACE inhibition activity was a result of the production of peptides from milk proteins due to their hydrolysis. Moreover, previous studies (*35*, *36*) reported that peptides produced by the fermentation of milk with different strains of microorganisms inhibited the activity of ACE.

### Antimicrobial activity of white rose flavoured soy yoghurt

Agar well diffusion assay was employed for determining the antimicrobial efficacy of unfermented soy milk and white rose flavoured functional soy yoghurt. The antimicrobial activity of all the products was evaluated against five major test organisms, *i.e. Escherichia coli, Staphylococcus aureus, Salmonella typhi, Listeria monocytogenes* and *Bacillus subtilis*. The zones of inhibition of the used indicator organisms ranged from 11.16 to 24.00 mm in diameter. White rose flavoured soy yoghurt had the highest antimicrobial activity against *Listeria monocytogenes* (24.00 mm), which was significantly higher (p<0.05) than others, namely *B. subtilis* (14.83 mm), *S. aureus* (12.00 mm), *S. typhi* (11.83 mm) and *E. coli* (11.16 mm), confirming *E. coli* as the most resistant (Fig. 2b). It can be assumed that the antimicrobial effect exerted by LAB in the yoghurt might be due to the reduction of pH caused by the production of lactic acid, acetic acid and other metabolites like diacetyl, hydrogen peroxide, fatty acids, aldehydes and other compounds (*39*). *Lactobacillus* spp. constitute the natural flora in the human gastrointestinal tract, and possess the ability to inhibit the harmful pathogens by causing an overall reduction in the intestinal pH, and thus inhibit the multiplication/binding of the pathogens in the intestinal mucosa (*40*).

### Total antioxidant activity of white rose flavoured soy yoghurt

Using ABTS radical scavenging method, the antioxidant activity of white rose flavoured functional soy yoghurt was estimated during different storage periods (0, 2, 5, 8 and 10 days) at 6 °C (Table 5). A wide range of antioxidant capacity was observable at different storage periods, *viz*. from 57.42 to 85.02% on day 0, from 57.57 to 86.02% on day 2, from 59.28 to 87.00% on day 5, from 52.28 to 82.06% on day 8 and from 41.14 to 78.12% on day 10. The ABTS reaction is the time-dependent. The variation in the antioxidant activity of white rose flavoured functional soy yoghurt among the different storage days was found to be highly significant (p<0.05). Generally, the protein hydrolysates present in the soy milk fermented by LAB contain several antioxidant peptides that have the potential to protect the human body by scavenging free radicals, such as reactive oxygen species, and also increase the shelf life of the fermented dairy foods by causing retardation of lipid peroxidation *via* hydrogen atom or electron transfer mechanisms (*37*). Reportedly, fermented soy milk and yoghurts produced using mixed *Lactobacillus* cultures showed a higher radical scavenging activity than the milk fermented using a single strain (*38*).

**Table 5:** Antioxidant activity of white rose flavoured soy yoghurt during storage

*t*(storage)/day)	*t*(scavenging)/s										
0	30	60	90	120	150	180	210	240	270	300
Antioxidant activity/%										
0	(57.42 ±0.04)^a^	(71.00 ±0.01)^c^	(74.00 ±0.06)^c^	(76.71 ±0.06)^c^	(78.57 ±0.04)^c^	(80.42 ±0.07)^f^	(81.71 ±0.08)^f^	(83.0 ±0.1)^f^	(83.85 ±0.01)^f^	(84.28 ±0.01)^f^	(85.02 ±0.02)^f^
2	(57.57 ±0.04)^a^	(72.14 ±0.02)^c^	(74.71 ±0.020)^c^	(77.85 ±0.02)^c^	(79.71 ±0.02)^c^	(81.42 ±0.02)^f^	(82.85 ±0.02)^f^	(83.42 ±0.01)^f^	(83.85 ±0.01)^f^	(84.85 ±0.01)^f^	(86.02 ±0.01)^f^
5	(59.28 ±0.04)^a^	(69.0 ±0.1)^d^	(74.85 ±0.04)^c^	(77.14 ±0.03)^c^	(80.00 ±0.03)^c^	(81.71 ±0.03)^f^	(82.71 ±0.04)^f^	(84.00 ±0.05)^f^	(84.71 ±0.04)^f^	(85.42 ±0.05)^f^	(87.00 ±0.05)^f^
8	(52.28 ±0.03)^a^	(69.42 ±0.04)^d^	(73.57 ±0.03)^c^	(76.42 ±0.02)^c^	(77.71 ±0.02)^c^	(78.71 ±0.02)^c^	(79.28 ±0.01)^c^	(79.85 ±0.01)^c^	(80.28 ±0.09)^c^	(81.00 ±0.08)^f^	(82.06 ±0.07)^f^
10	(41.14 ±0.03)^b^	(56.14 ±0.05)^a^	(61.28 ±0.06)^d^	(64.85 ±0.06)^d^	(67.00 ±0.06)^d^	(68.85 ±0.07)^d^	(70.14 ±0.07)^d^	(71.28 ±0.07)^c^	(72.14± 0.07)^c^	(72.85 ±0.07)^c^	(78.12 ±0.06)^c^

Values are mean±S.D. of three independent determinations of each sample. Values with different letters in superscript in each column differ significantly (p<0.05)

### Biotransformation of isoflavones

**Fig. 3:**
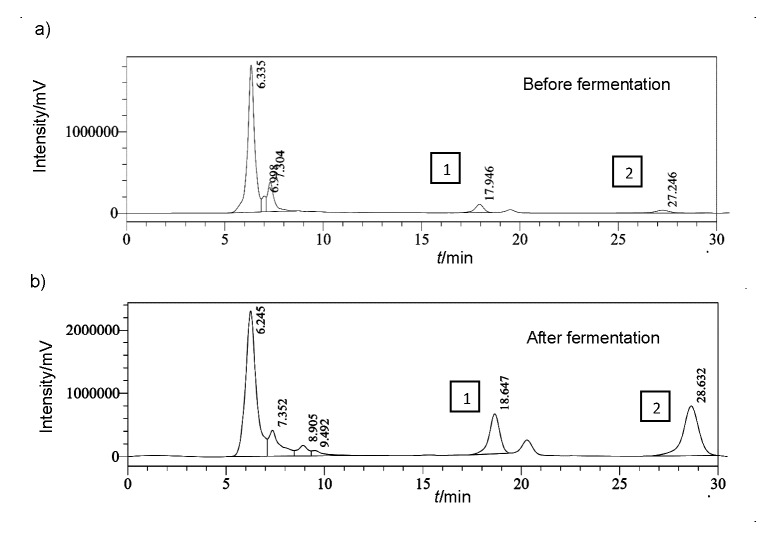
Biotransformation of isoflavones in: a) unfermented soy milk [1=daidzein (34.4%), 2=genistein (30.6%)], and b) fermented white rose flavoured soy yoghurt [1=daidzein (58.4%), 2=genistein (87.3%)]

Soy foods contain a considerable amount of isoflavones that exists as a complex mixture of glucoside conjugates. The isoflavones genistin, daidzin, genistein and daidzein were successfully separated with RP-HPLC from functional soy yoghurt after an incubation period of 24 h. The most abundant form of isoflavones in soy milk are glycosides (daidzin and genistin), which are converted to the corresponding aglycones (daidzein and genistein) during the fermentation of soy milk. The amount of glycosides (daidzin and genistin) was 34.4 and 30.6%, respectively, and the amount of their corresponding aglycones (daidzein and genistein) after the fermentation was 58.45 and 87.3%, respectively (Fig. 3). There was a significant decrease in the amount of glucosides and a considerable increase in the amount of aglycones after fermentation. A possible interpretation can be that *Lactobacillus* culture improved the bioconversion of isoflavones in fermented soy yoghurt as compared to unfermented soy milk. Otieno *et al*. (*21*) reported a significant (p<0.05) increase in the concentration of individual aglycones (daidzein, glycitein and genistein) in around 12 h when using *L. acidophilus* 33200, *L. acidophilus* 4962, *L. acidophilus* 4461 and *L. casei* ASCC 290, while it took 24 h for a significant increase (p<0.05) in aglycones when using *B. animalis* Bb12 and *L. casei* 2607. In the soy product supplemented with isoflavones, the aglycone forms were predominant and the amount of daidzein was the highest (77.8%, *i.e.* 32.62 mg/100 g) (*41*). Tsangalis *et al.* (*42*) used ruptured cells of *B. animalis* Bb12, *B. pseudolongum* and *B. longum*, resulting in a maximum increase in the concentration of aglycones after incubation for 24 h.

## CONCLUSION

We can conclude that when fermented with strongly probiotic lactic acid bacteria, soy milk can be a rich source of bioactive antioxidants with potent ACE inhibitory peptides. The functional white rose flavoured soy yoghurt development in this study had antagonistic activity against several major indicator organisms. Isoflavones like genistin, daidzin, genistein and daidzein, with high estrogenic effects, were successfully detected by RP-HPLC in the yoghurt samples. In conclusion, the *Lactobacillus* strains used in our study can be applied for the development of various functional fermented soy foods enriched with aglyconic forms of isoflavones and ACE inhibitory peptides, with potential probiotic and therapeutic attributes.
